# Observations on Chronic Hepatitis

**Published:** 1812-10

**Authors:** 


*To the Editors of the Medical and Physical Journal.*

GENTLEMEN,

AMONG the numerous contributors to your valuable Journal, no one is more conspicuous than Mr. Fogo. This indefatigable gentleman has honored many of your pages with his ingenious criticisms, and done every thing in his power to set right those blunderers whose productions you have permitted to occupy a place in your volumes. If the instruction which he so liberally affords on every occasion meet not with that attention which his labors give him reason to expect, we can only attribute it to perverseness of disposition, and that obstinacy in human nature which rejects advice when gratuitously offered.

I have neither leisure nor inclination to search your volumes for the various productions of this gentleman, but if I am not mistaken, they are all written in the same strain. I shall, therefore, only take the liberty of making a few remarks on his last communication. Indeed, I intended to have noticed his paper on twin cases, inserted in your Journal for May last; but, as a reply has already appeared in your present Number, I shall postpone any remarks, only observing, that, if several passages in it are highly indecorous, in his present communication there are many more extremely offensive. *Acquirit vires eundo*, is not inapplicable to him in the present instance.

What has now, professedly, called forth his critical powers, is Dr. Thompson's case of abscess of the liver, in your 158th Number. This gentleman's case appears to be written with all that unassuming modesty which characterises real genius. He first states the symptoms, next gives his indications of cure, and the reason why he prefers the means he used to any other; and, lastly, gives his own opinion on the functions of the liver, which he modestly styles mere hypothetical conjectures. One would have thought that this acknowledgment was sufficient to have disarmed the rage of criticism, or rather, from the tenor of the whole communication, left no room for the critic to wield his pen. The only publication the paper could have, with propriety, given rise to, is a systematic arrangement of the diseases of the liver, with the symptoms by which they may be known and distinguished; for which, Dr. T. says, he would be grateful, and I believe many more besides him.

Let us now attend to what Mr. Fogo says on the subject. After repeating Dr. T.'s statement of the symptoms, he tells us, "this is a genuine case of chronic hepatitis, allowed from bad or no treatment to arrive at suppuration;" and, in a line a line or two after, says, "but, when it is considered that the disease is not understood by many of the profession, it is of less consequence, as he would have got no medicines necessary to remove the obstruction of the biliary or other vessels in the liver, in the early stage of the disease." I rather think that Mr. F. has here made a small mistake, by saying, not understood by *many* of the profession, and that he intended to write—not understood by *any* of the profession; else why might not the patient have been fortunate enough to have applied to one of those professional gentlemen who, perhaps, understood the disease *nearly* as well as Mr. F. himself? Be that as it may, I should be glad to know where Mr. F. derives his information that chronic hepatitis is not understood by many of the profession. As we have only his bare assertion for the fact, I have no hesitation in asserting the direct contrary, though anonymous, and that chronic hepatitis is as well known, and the treatment as generally understood, as any other disease of so frequent occurrence. I believe Mr. F. would have some difficulty in finding, among the most inexperienced practitioners in this country, one so ignorant who could not tell him, if a patient complained of a dull obtuse pain in the right hypochondrium, with dyspepsia, &c. &c. that the disease was seated in the liver.

Dr. T., says Mr. F., may perhaps be surprised at the name of chronic hepatitis, &c. &c. Now, we cannot think that the ignorance of this physician is so great as Mr. F. would have us believe. In attentively perusing his paper, it is evident he formed a correct opinion of the case from the time of the patient's first application, as he perspicuously lays down the indications of cure; and adds, "but I preferred emetics, because I thought they would effect that purpose quicker, and with more certainty; and because I considered the action of vomiting was likely to empty the *abscess* more completely than could be done by any other means."

After bestowing some reflections on Dr. Cullen, and his annotator, Dr. Rotherham, not forgetting the ignorance under which the hearers and readers of the former author must labor, he discovers that Dr. Cullen must have read many books; but, not having met with any regular description of the disease, &c. tells us, that this proves that there is no information to be got in any book, either ancient or modern; "this also shows (he continues) that there is a great chasm in the system of physic; that the largest organ in the body, and of the most essential use and importance, should

I have have been and still is forgot." And, a little further on, he says, "the chronic diseases of that gland are not only overlooked, but the gland itself is scarcely thought of; and, in the apparent sentiments of both ancients and moderns, it seems of no more consideration, than a large body hung up at the top of the abdominal viscera, for the purpose of filling up the cavity." Where Mr. F. learned his logic I know not; but I conceive he draws inferences not warranted by the premises. Because Dr. Cullen has done little more than mention chronic hepatitis, and Dr. Rotherham has "lulled the profession still more into a fatal blindness, ignorance, and security, by telling them it is doubtful whether this chronic hepatitis ever exists;" is it thereby proved, that there is no information to be got in any book, either ancient or modern, or that the disease is not understood by many of the profession?

Whether chronic diseases of the liver are not only overlooked and the gland itself scarcely thought of, we shall presently see. As to the witticism about the large body hung up at the top of the abdominal viscera, we shall leave Mr. F. in the full enjoyment of it. Had Mr. F. deigned to read more, he perhaps would have hesitated before he had committed himself in the present instance. So far from the liver and its diseases being neglected, more has, perhaps, been written expressly on the subject, and more attention given to that gland, than to almost any other part of the system. In order to show whether Mr. F.'s assertions be correct, I will merely mention a few of the authors to whom he may refer: Dr. Pringle on Diseases of the Army; Dr. Fordyce's Elements, part 2; Dr. Brook Mathews on Hepatic Diseases; Dr. Saunders on the Liver; London Practice of Physic; Nisbet's Clinical Guide; Dr. Pemberton on the Abdominal Viscera; Hooper's Physician's Vade Mecum, &c. &c. He will find in many of these authors a detailed account of the symptoms of chronic hepatitis, and that it is more frequent in this country than the acute species; and also, that the remedies most to be relied upon in the cure are, mercury, the nitric acid, and a continued course of bitter tonics and aperients.

It would be tedious and unprofitable to follow this writer through the whole paper: I hope your pages will be better occupied than with either Mr. F.'s criticisms or my animadversions: I shall, therefore, only notice one or two more passages. Mr. F. seems extremely jealous of any person knowing any thing about the diseases of the liver but himself; as he observes, "I doubt, from Dr. T.'s vacillating opinion, opinion, &c. he might not have discovered the disease in its early stage," &c. Dr. T. does not vacillate in his opinion, he only doubts if the liver is not designed for more purposes than we are at present aware of; and, as to the disease itself, it is clear he had formed a competent idea of its nature from the patient's first application to him. Mr. F. is now led to expatiate on the great ignorance of the three gentlemen who attended the patient in the late Dr. Paxton's case. It was natural, says he, for these three practitioners to neglect the pain in the side, from never having heard that such a disease as chronic hepatitis ever existed. This gentleman never fails to assert the ignorance of his professional brethren, whether well founded or otherwise. There is nothing in Dr. Paxton's paper to lead to so unfair a conclusion. Medical knowledge must be at a low ebb, indeed, when a physician, and two other practitioners, never heard that so common a disease as chronic hepatitis ever existed. Dr. P. and his partner not coinciding in opinion with the physician, is no ground for Mr. F.'s assertion.

The concluding sentences of this gentleman's communication are not unworthy of notice. "These (says he) are the unblessed effects of our teachers and writers, and all before them," &c. And again, "These two cases, had there never been another, ought to be an awful warning to the profession," &c. &c. He then finishes, by informing us that the disease is very common in his neighbourhood; that a week seldom passes without one or more patients applying for advice; and in many hundred cases he has been able to detect the obscure disease in its early stage, and been fortunate to remove the obstruction before it arrived at suppuration, or formed any other disease.

Has Mr. F. yet to learn that abscess of the liver is by no means a frequent occurrence in this country, although chronic hepatitis is a very common disease? Setting aside the cavalier manner in which Mr. F. treats the profession, both generally and individually, all the information to be obtained from his paper amounts only to this—That, from Hippocrates down to the present time, no person has known any thing about chronic hepatitis, or hepatic diseases in general, except Mr. A. Fogo, of Newcastle, who has been fortunate enough to detect the obscure disease, and arrest its progress in many hundred cases.

I am, Gentlemen,

Your humble Servant,

*August* 15, 1812. H——.

COLLEC-

